# An Idiopathic Hepatic Hematoma Presenting in the Neonatal Period

**DOI:** 10.7759/cureus.110252

**Published:** 2026-06-04

**Authors:** Lamya Eliaziji, Ilham Elouardighi, Amina Barkat

**Affiliations:** 1 Research Team on Health and Nutrition of Mother and Child, Faculty of Medicine and Pharmacy, Mohammed V University in Rabat, Rabat, MAR; 2 Pediatrics, Hôpital d'enfant de Rabat, Rabat, MAR

**Keywords:** hemoperitoneum, hepatic hematoma, liver injury, newborn, ultrasound

## Abstract

Hepatic hematoma in newborns is an extremely rare condition, usually secondary to birth trauma or a vascular malformation. Clinically, it is often asymptomatic but can lead to serious complications, including internal bleeding and even signs of liver failure. We report an unusual case of intraparenchymal hepatic hematoma of undetermined etiology in a newborn, without associated birth trauma. This is a male newborn, born at term, admitted on the third day of life for respiratory distress associated with pallor of the skin and mucous membranes and abdominal distension with scrotal and inguinal ecchymoses. An abdominal ultrasound was performed, revealing hepatic hematoma. An abdominal CT scan was subsequently performed, showing a heterogeneous, liquefied intrahepatic collection with a hemorrhagic mass and hyperdense infiltrates in the iliac fossae. Hepatic hematoma is a poorly understood condition with potentially extremely serious consequences, particularly because it can occur idiopathically without a known cause. Indeed, given that clinical symptoms are sometimes nonspecific and that laboratory results may only become apparent later, diagnosis relies primarily on ultrasound, and treatment is most often conservative.

## Introduction

Hepatic hematoma in newborns is a rare condition, typically caused by obstetric trauma or vascular malformation. This condition often occurs due to injury to the hepatic vessels or the hepatic capsule during delivery [1]. Although frequently asymptomatic, hepatic hematoma can lead to serious complications, such as internal bleeding, jaundice, or signs of liver failure. It is usually subcapsular. In some cases, hepatic hematoma may be associated with liver rupture and/or hemoperitoneum [2,3]. Several causes have been described, including obstetric trauma, coagulopathies, and neonatal sepsis [4].

Here, we report an unusual case of a newborn with an intraparenchymal hepatic hematoma of unknown cause, with no history of obstetric trauma.

## Case presentation

This is a full-term male newborn who was admitted on day three of life for respiratory distress accompanied by pallor of the skin and mucous membranes. The pregnancy was monitored, proceeded normally, and was carried to term. The mother is 20 years old, with no significant medical history. There is no history of consanguinity or obstetric trauma. She did not take any medications throughout the pregnancy.

Medical history revealed no history of hemophilia in the family.

The delivery was a vaginal birth without instruments; the Apgar score was 10/10/10, and the birth weight was 3600 g. Vitamin K has been received, and the umbilical catheter has not been inserted.

**Figure 1:**
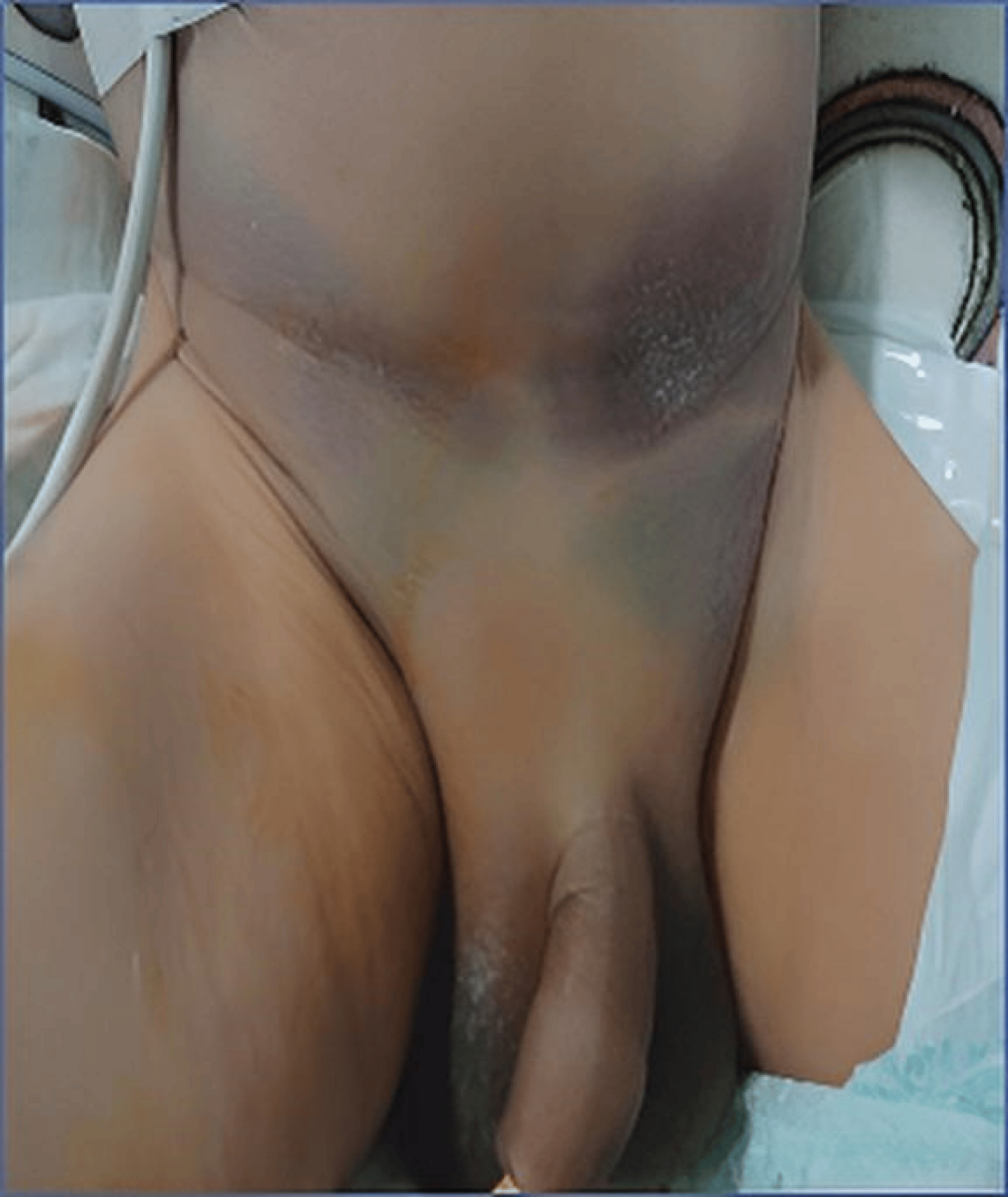
Bruises in the inguinal folds

**Figure 2:**
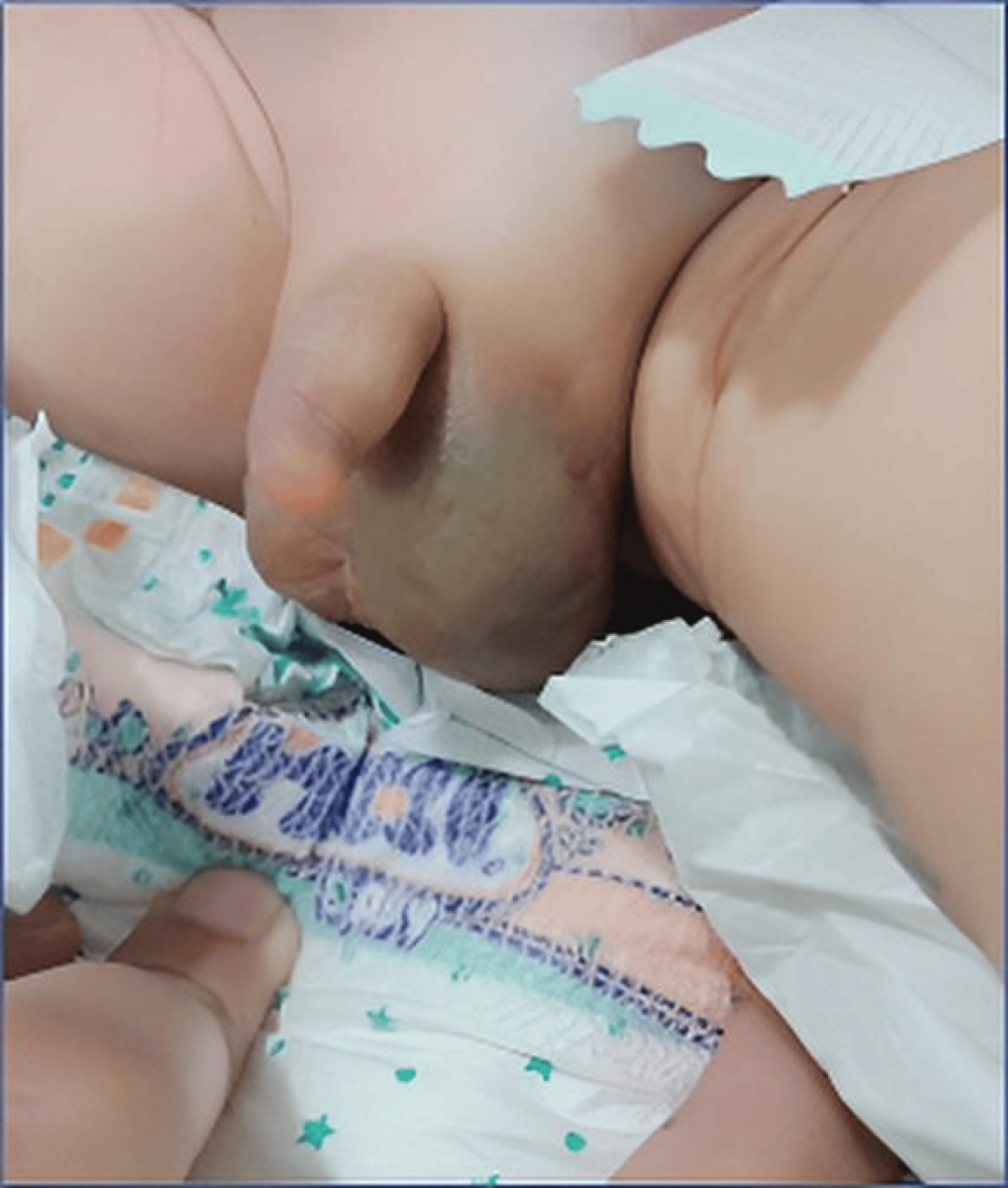
Bruises on the scrotum

The clinical examination revealed a pale, hypotonic, responsive newborn who was hemodynamically and respiratory stable. Examination of the abdomen and external genitalia revealed abdominal distension, with a tender liver span of 10 cm and a subcostal margin of 4 cm, and bruising in the scrotum and inguinal folds (Figures 1, 2).

**Figure 3:**
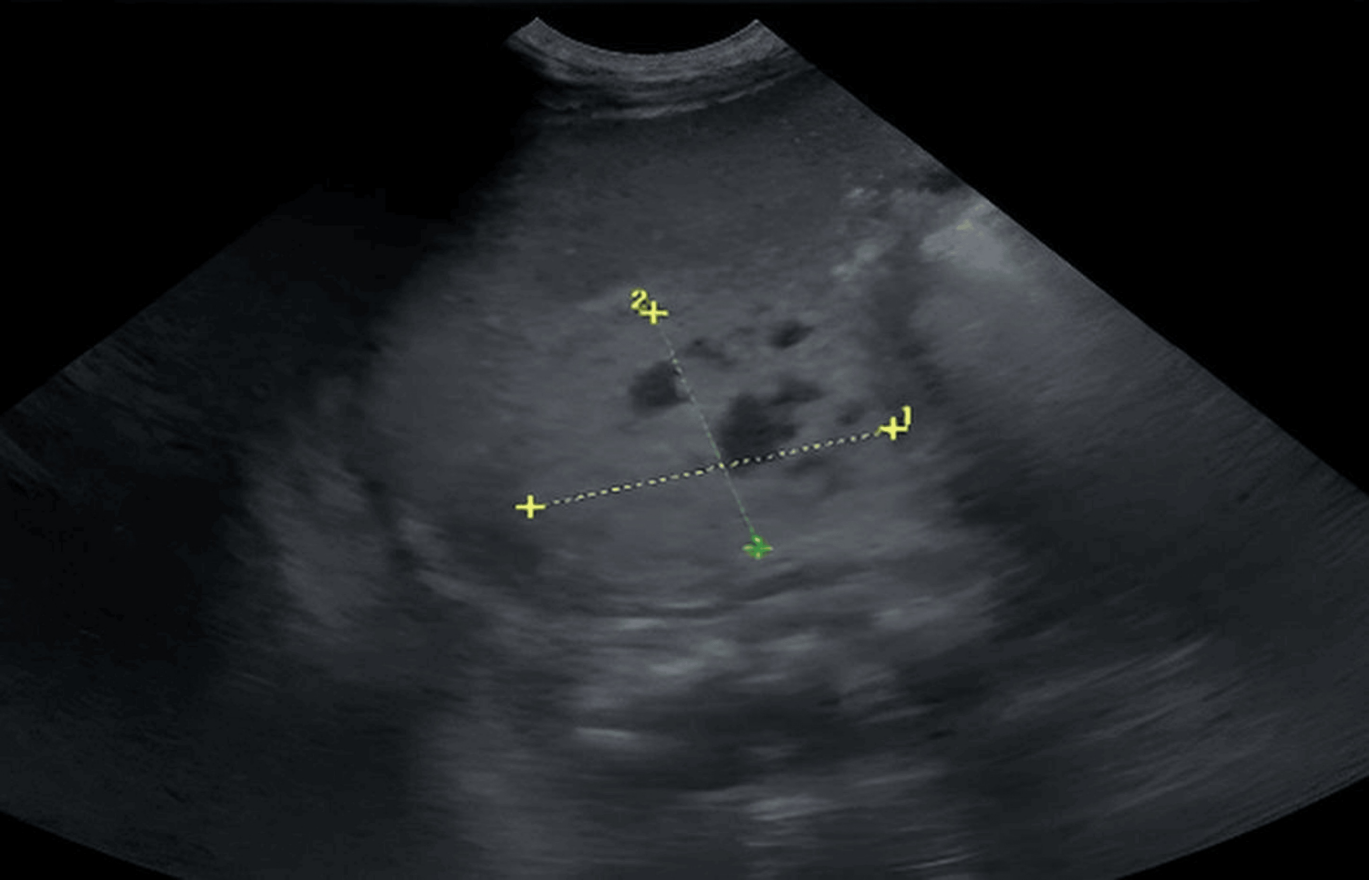
Ultrasound image of a hepatic hematoma in our patient

**Figure 4:**
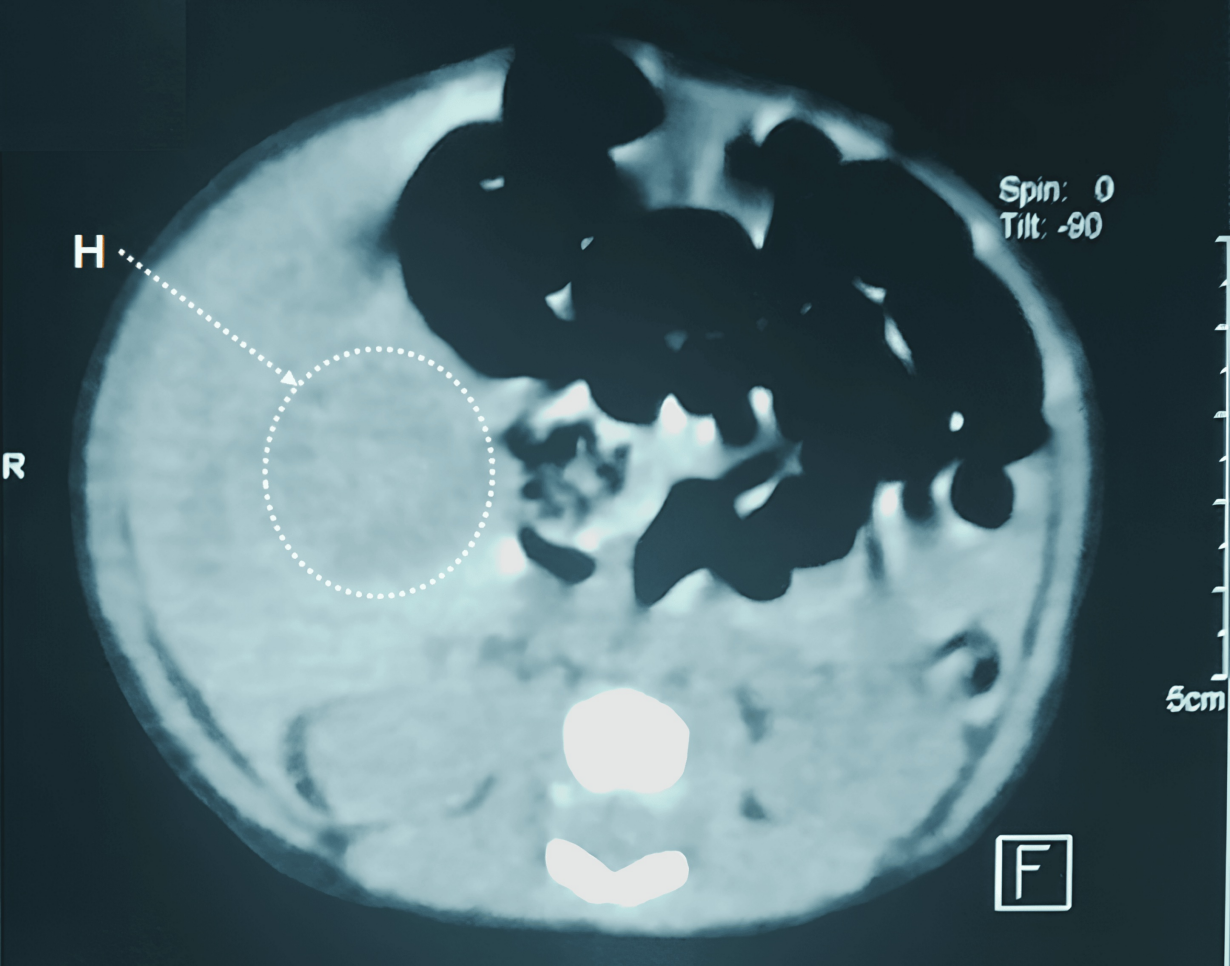
Abdominal-pelvic CT scan showing a hepatic hematoma (H: Borders of the hematoma)

**Figure 5:**
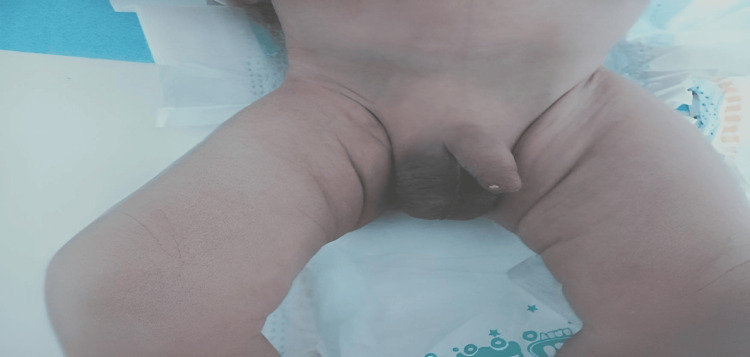
Resolution of bruises

An abdominal ultrasound was ordered, revealing an intraparenchymal hepatic hematoma (Figure 3); this was supplemented by an abdominal computed tomography (CT) scan, which showed a heterogeneous liquefied intrahepatic collection affecting segments VI and VII, with a hemorrhagic tumor and hyperdense infiltrates adjacent to the iliac fossae measuring 37 x 35 mm. An EUS was ordered to rule out secondary sites, with no abnormalities found (Figures 4, 5).

Laboratory test results revealed severe anemia at 5 g/dL with thrombocytopenia at 71,000 platelets/mL, requiring red blood cell and platelet transfusions. A hemostasis panel was performed prior to transfusion, with coagulation factor levels found to be normal. Renal and hepatic function tests were normal. C-reactive protein (CRP) was negative at 3.2 mg/L.

The clinical course was favorable, marked by the regression of ecchymoses and the hepatic hematoma on follow-up ultrasounds. At three months of age, the follow-up ultrasound showed its complete resolution.

## Discussion

Hepatic hematomas are rarely diagnosed in newborns, except during perinatal autopsy, and are, in most cases, subcapsular [2]. Various causes have been implicated in these hematomas, such as trauma, sepsis, and coagulopathies [3]. The intraparenchymal location and idiopathic nature of this lesion distinguish our case from those previously reported. Liver trauma should be suspected in any newborn with a predisposing risk factor, whether maternal, fetal, or related to delivery, as well as in cases of acute anemia or shock of uncertain etiology. This is a potentially fatal condition, and early detection remains the best way to reduce complications [4]. The clinical presentation of neonatal hepatic hematoma is nonspecific and severe, depending on the extent of blood loss, which can lead to hypovolemic shock. Hepatic hematomas may present insidiously with progressive anemia, jaundice, irritability, or respiratory distress. They rarely present as an isolated abdominal mass and may mimic a liver tumor. However, they can gradually increase in size until they rupture, leading to acute deterioration. A scrotal hematoma has been described as a telltale sign [4]. In our case, the patient presented with marked pallor; bilateral scrotal hematomas; inguinal hematomas; and tenderness in the right upper quadrant. Abdominal ultrasound is the test of choice for diagnosing hepatic hematoma; it allows for precise visualization of the lesion, enables differential diagnosis with neoplastic liver disease, rules out rupture into the peritoneal cavity, and facilitates long-term follow-up [5]. Abdominal CT is debatable, but it can better assess liver lesions detected by ultrasound and determine their extent and age [6]. Neonatal hepatic hematoma may be a warning sign of a potential underlying coagulopathy, such as DIC and coagulation factor deficiencies, requiring prompt investigation. These coagulopathies create a predisposition to bleeding, making the liver vulnerable. Rare cases of hemophilia diagnosed following hepatic hematoma in the newborn have been reported in the literature [7,8]. Treatment is primarily conservative; it consists of correcting the hemodynamic status and any associated coagulation disorder. In cases of rupture or hemodynamic instability unresponsive to treatment, a laparotomy is necessary to control the bleeding [3]. Hepatic hematomas should be suspected in any newborn presenting with acute anemia or hypovolemic shock of unknown cause.

## Conclusions

Hepatic hematoma is a relatively rare condition that can be extremely serious and is likely of idiopathic origin. Given the often-nonspecific clinical symptoms and delayed test results, diagnosis relies primarily on ultrasound, and treatment is often conservative.
